# Debranched Lentil Starch–Sodium Alginate-Based Encapsulated Particles of *Lacticaseibacillus rhamnosus* GG: Morphology, Structural Characterization, In Vitro Release Behavior, and Storage Stability

**DOI:** 10.3390/foods13244047

**Published:** 2024-12-15

**Authors:** Jinxiu Zhang, Xinzhong Hu, Zhen Ma

**Affiliations:** College of Food Engineering and Nutritional Science, Shaanxi Normal University, Xi’an 710062, China; zh15838526998@163.com (J.Z.); hxinzhong@snnu.edu.cn (X.H.)

**Keywords:** debranched starch, molecular structural characteristics, *Lacticaseibacillus rhamnosus* GG, encapsulation

## Abstract

Starches with different degrees of debranching (DBS30, DBS60, and DBS90) and sodium alginate were used as the wall material for encapsulating particles of *Lacticaseibacillus rhamnosus* GG (LGG). The structural characteristics of these encapsulated particles were examined, along with the impact of varying levels of debranching on the encapsulation efficiency, the in vitro release of LGG under the simulated gastrointestinal environment, and the storage stability of the encapsulated particles. The results revealed a transformation in the crystalline polymorph from C- to B+V-type following debranching and retrogradation. This process also resulted in a significant decrease in molecular weight and polydispersity index, accompanied by an increase in amylose and resistant starch levels along with the relative crystallinity of the debranched lentil starch. Comparatively, DBS60-LGG and DBS90-LGG exhibited higher encapsulation efficiency and encapsulation yield than UDBS-LGG and DBS30-LGG. Furthermore, these encapsulated particles provided enhanced protection for LGG in both the simulated gastrointestinal environment and the storage process. It can be inferred that a superior encapsulation performance of the debranched lentil starch–sodium alginate-based encapsulated LGG particles was associated with higher debranching levels, a more uniform molecular weight distribution, and a more ordered multi-scale structure of the debranched lentil starch.

## 1. Introduction

*Lacticaseibacillus rhamnosus* GG (LGG) is well known for its diverse probiotic functions, such as regulating intestinal microbiota balance [1], enhancing host lipid metabolism [2,3], and improving overall body immunity [4]. However, the probiotic viability of LGG is constrained by factors such as gastric acid, bile salt, oxygen, and high temperature. In order to overcome these limitations, researchers have dedicated their efforts towards the advancement of encapsulation as a promising delivery system [5,6], aiming to improve the biological activity and stability of functional probiotic ingredients in foods, while also facilitating controlled release over a desired duration in the gastrointestinal tract.

Synthetic polymers should be cited as alternatives to natural ones for the encapsulation and delivery of probiotics, yet their use is often limited by restricted solubility in water. This challenge can be mitigated by a novel class of amphiphilic polymers designed to self-assemble in aqueous environments [7]. Starch, a biodegradable polymer with excellent biocompatibility, is widely used in the construction of functional encapsulated systems due to its cost-effectiveness. Nevertheless, the lengthy molecular chains found in native starch make it susceptible to gastric acid and amylase, thereby limiting its effectiveness as an encapsulation wall material. To enhance protection and enable the controlled release of functional probiotics, researchers have explored the selective hydrolysis of α-1,6 glycosidic bonds in starch chains through physical, chemical, and enzymatic treatments. This process results in yields of linear debranched starches with a reduced molecular weight, which can be further recrystallized to enhance enzymatic resistance [8,9,10]. Lentil seeds (*Lens culinaris*) contain approximately 60% starch [11]. The uniquely high ratio of amylose to amylopectin in lentil starch contributes to its low crystallinity and gelatinization enthalpy. Compared to corn and potato starch gels, lentil starch gels exhibit a superior storage modulus, gel strength, and pasting viscosity. As a result, lentil starch is ideal for applications requiring enhanced gel strength and pasting viscosity [12]. The low glycemic index of lentil starch also helps lower the glycemic response in humans, making it a good option for individuals with obesity and diabetes.

Pullulanase, an enzyme specifically targeting the α-1,6 glycosidic bond in starch substrate, is commonly used for debranching starch. Debranched starch (DBS) obtained via pullulanase treatment exhibits improved thermal stability and a higher content of slowly digestible starch and resistant starch (RS) in comparison to native starch [13]. Sodium alginate (SA) is frequently utilized for encapsulating probiotics by forming a gel state through cross-linking with calcium ions. Sodium alginate serves as a natural polymer capsule carrier, while calcium chloride acts as an ion bridge [14]. SA contains functional groups, including carboxyl and hydroxyl groups, making it an ideal carrier for natural polymer-encapsuled particles. However, encapsuled particles composed solely of sodium alginate often face challenges such as physical instability, porosity, and a structure with hydrophilicity [15]. These factors make the encapsulated probiotics susceptible to adverse environments [16]. Studies have provided evidence that encapsulated particles of functional probiotics, which utilize a combination of sodium alginate and starch, such as those containing *Lactobacillus plantarum* ATCC 8014 and *Bifidobacterium bifidum* ATCC 1903, maintain high viability during storage [17]. Similar results were documented by Martin et al. [18] and Oberoi et al. [19], who employed a combination of starch and sodium alginate for probiotic encapsulation. Furthermore, a growing interest in utilizing debranched starch as a wall material for encapsulated particles has developed recently. Different degrees of debranching in DBS can influence the retrogradation patterns of short glucan chains, the crystalline structure, the morphological characteristics, and the hydrogel properties of DBS, which could affect its potential use as the delivery carrier of LGG probiotics [20,21,22]. However, limited research currently exists on how varying the degree of debranching affects the encapsulation efficacy and the stability of encapsulated probiotic particles.

Therefore, this study attempts to obtain debranched starch with different debranching degrees and employ it with sodium alginate as the wall material for encapsulated LGG particles, aiming to enhance the viability of LGG and protect it from detrimental factors such as pH, oxygen, temperature, and digestive fluids during the processing, transportation, storage, consumption, and marketing of probiotic products. By doing so, it addresses the current limitations hindering the widespread use of LGG, while also enabling controlled release within the gastrointestinal tract over a desired duration. The structural characteristics of the encapsulated particles are preliminarily assessed, and the impacts of varying debranching degrees on the encapsulation rate, the tolerance of encapsulated LGG in a simulated gastrointestinal environment, and the storage stability of the encapsulated particles are further examined, and the potential relationship between the structural features of DBS carriers and their release behavior was further evaluated. The findings are expected to introduce a novel approach for the preparation of encapsulated LGG particles as well as to provide a theoretical foundation for the application of debranched starch in probiotic encapsulation technology.

## 2. Materials and Methods

### 2.1. Materials

The lentil seeds were provided by Gansu Qichen Agricultural Products Co. (Baiyin, Gansu Province, China). *Lacticaseibacillus rhamnosus* GG (LGG, ATCC 53103) was sourced from American Type Culture Collection (ATCC, Manassas, VA, USA). Pullulanase (E.C. 3.2.1.41, ~1498 NPUN/g) and dextran were purchased from Sigma-Aldrich Chemical Co. (Saint Louis, MO, USA), acetate buffer, phosphate buffer, and MRS broth were obtained from Fisher Scientific Inc. (Pittsburgh, PA, USA). The RS assay kit and amylose/amylopectin assay kit were provided by Megazyme International Ireland Ltd. (Wicklow, Ireland). The sodium alginate, calcium chloride, sodium chloride, potassium bromide, saline, sodium cyanoborohydride, glycerol, pepsin, bile salt, and pancreatic enzyme used were of biochemical grade and were supplied by Shanghai Aladdin Reagent Co. (Shanghai, China). HPLC-grade DMSO and LiBr were supplied by Tedia (Fairfield, OH, USA). All other reagents and chemicals utilized in this study were of analytical grade.

### 2.2. Preparation of Debranched Lentil Starch (DBS) with Short Chain Length

Native starch was isolated from lentil seeds according to the approach outlined in our earlier research [23]. A starch slurry was prepared by dispersing native lentil starch in distilled water at a concentration of 20% (*w*/*v*), followed by pre-gelatinizing in boiling water for 15 min and pressure cooking at 121 °C and 0.1 MPa for 30 min in an autoclave (LDZX-30KBS, Shanghai Shenan Medical Instrument, Shanghai, China). Subsequently, acetate buffer (200 mmol/L, pH 5.5) was added in equal volume and dispersed. After cooling to 55 °C, pullulanase was added at 30, 60, and 90 NPUN/g of starch, the mixture was then incubated at 55 °C for 8 h with constant stirring. The debranching conditions were selected based on a previous study [24] and our preliminary investigations, wherein the amylose content was relatively high (>50%), corresponding to the effective extent of starch debranching. To inactivate the enzyme activity, starch samples were boiled for 15 min immediately, and the supernatant was collected through centrifugation (2504× *g*, 10 min). The obtained supernatant was then divided into two segments: one segment was recrystallized at 4 °C for 24 h, freeze dried for 48 h in a freeze dryer (Lab-1A-80E, Boyikang Instrument Co., Ltd., Beijing, China), and sieved through a 149 μm sieve to prepare debranched lentil starch (DBS), whereas the remaining supernatant was used for the preparation of encapsuled LGG particles. The resulting debranched lentil starch samples were labeled DBS30, DBS60, and DBS90, and prepared with pullulanase at a concentration of 30, 60, and 90 NPUN/g, respectively. The unbranched lentil starch (UDBS) was used as the control group for further analysis as a native starch undergoing pre-gelatinizing and pressure cooking at 121 °C and 0.1 MPa for 30 min without the debranching treatment.

### 2.3. Preparation of LGG Bacterial Suspensions

The stored LGG strains were thawed from −80 °C and activated twice in MRS broth (2.0% *w*/*v*) at 37 °C for 24 h under anaerobic conditions. Subsequently, the activated LGG was incubated in MRS broth for 12 h until it reached the final stage of logarithmic growth, with the maximum viable bacteria count. The harvesting of the LGG was carried out by centrifugation (Velocity 14R, Dynamica, London, UK) at 4 °C and 7100× *g* for 10 min. The resulting sediment was then subjected to multiple washes with sterile NaCl (0.9%) and was resuspended in NaCl (0.9%). The LGG suspension was accurately quantified using the plate counting method and stored at 4 °C for subsequent experiments.

### 2.4. Preparation of Encapsulate LGG Particles

**Figure 1:**
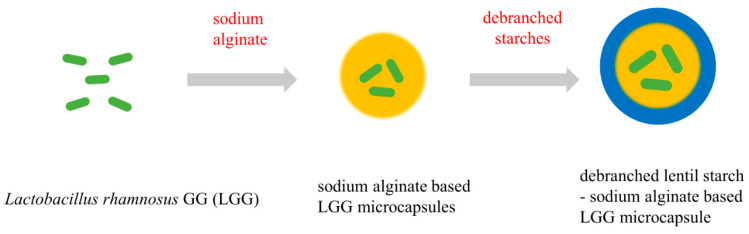
Visual diagram of the encapsulation process of LGG.

The preparation of the encapsulated LGG particles was conducted following the procedure outlined by Morsy et al. [25] and Oberoi et al. [19] with slight modification. Firstly, 10 mL of LGG suspension (1.43 × 10^8^ CFU/mL) was thoroughly blended with 90 mL of sterile sodium alginate solution (2%, *w*/*v*). Then, the sterilized calcium chloride solution (0.05 mol/L) was added dropwise under continuous stirring, and the mixture was continuously stirred at 25 °C for 30 min to form a gel. Subsequently, the supernatants of UDBS, DBS30, DBS60, and DBS90 were immediately added, followed by continuous stirring for 1 h and incubation at 4 °C for 24 h for retrogradation. A visual diagram of the encapsulation process is shown in Figure 1. The encapsulated LGG particles were obtained by freeze drying and were referred to as UDBS-LGG, DBS30-LGG, DBS60-LGG, and DBS90-LGG.

### 2.5. Determination of the Content of Amylose and RS

The amylose and RS content of UDBS, DBS30, DBS60, and DBS90 was determined using the amylose/amylopectin assay kit and resistant starch assay kit based on the manufacturer’s guidelines.

### 2.6. High-Performance Size-Exclusion Chromatography (HPSEC)

The molecular weight distribution of the freeze-dried samples of debranched starch was analyzed using an HPSEC system (Agilent 1260 series SEC system, Agilent Technologies, Waldbronn, Germany) equipped with a multi-angle laser light-scattering (MALLS, DAWN-HELEOS II, Wyatt Technology Corp., Santa Barbara, CA, USA) detector as described by Zhou et al. [26]. The starch samples were dissolved in a dimethyl sulfoxide (DMSO) solution containing 0.5% LiBr, stirred at 80 °C for 16 h, and then centrifuged at 8000× *g* for 10 min. The supernatant was filtered (0.45 μm) and injected into the chromatographic column, which was eluted with a DMSO solution at a flow rate of 0.3 mL/min. Calibration was performed using dextran with known molecular weight. The dextran samples with different molecular weights (5 kDa, 40 kDa, and 200 kDa) were used for the calibration curve (R^2^ = 0.9731). The weight average molecular weight (*M_w_*) and polydispersity index (PDI = *M_w_*/*M_n_*) were determined using Wyatt Astra software (V 6.1.4.25).

### 2.7. X-Ray Diffraction (XRD)

The crystal structural characteristics of the freeze-dried DBS and encapsulated LGG particles were analyzed using a D/Max2550 X-ray diffractometer (Rigaku Co.™, Tokyo, Japan) equipped with a Cu-Kα radiation detector. The samples were continuously scanned through the 2θ range between 4° and 40° with a step size of 0.02 and a scanning rate of 5°/min. The relative crystallinity (C1) was calculated using Jade software (V 5.0, Materials Data Inc., Livermore, CA, USA) following the method described by Yin et al. [27].

### 2.8. Solid-State ^13^C Cross-Polarization and Magic Angle Spinning Nuclear Magnetic Resonance (^13^C CP/MAS NMR)

The freeze-dried samples (200 mg) were scanned using a solid-state ^13^C CP/MAS NMR spectrometer (AVANCE III 400 MHz WB spectrometer, Bruker Inc., Bremen, Germany) with a double-resonance H/X CP-MAS 4 mm probe at a frequency of 100.62 MHz and a spectral width of 40 kHz. The amorphous starch was obtained by heating native lentil starch suspensions (5% *w*/*v*) at 95 °C for 30 min, and then lyophilizing them immediately. The relative crystallinity (C2), double-helix content, and the proportion of the amorphous phase (PPA) were estimated using Peakfit software (V 4.12, SeaSolve, San Jose, CA, USA).

### 2.9. Fourier-Transform Infrared Spectroscopy (FT-IR)

The freeze-dried DBS and encapsulated LGG particle samples were mixed with KBr at a ratio of 1:100 (g/g). The mixture was thoroughly ground and pressed into a sheet. The FT-IR spectra of the samples were recorded using a Fourier-transform infrared spectrometer (TENSOR27, Bruker Optics GmBH, Ettlingen, Germany) in the spectral range of 400–4000 cm^−1^ with a resolution of 4 cm^−1^.

### 2.10. Scanning Electron Microscope (SEM)

For the SEM analysis, a few freeze-dried powder samples were fixed on copper stubs using double-sided conductive tape and then sputter-coated with gold. The morphological images of the samples were captured using a scanning electron microscope (Quanta 200 FEI Company, Hillsboro, OR, USA) at magnifications of 2000× and 10,000×, respectively.

### 2.11. Confocal Laser Scanning Microscope (CLSM)

For the CLSM analysis, starch samples and the encapsulated particle samples (10 mg) were dispersed in 15 μL of 1 M sodium cyanoborohydride, 15 μL of 20 mM 8-amino-1,3,6-pyranotrisulfonic acid (APTS) was then added to the dispersion. The mixtures were incubated at 30 °C for 18 h, washed repeatedly with distilled water, and centrifuged at 10,000× *g* rpm/min for 5 min. The samples were suspended in 50% glycerol, and the fluorescence signals were observed at 488 nm using a confocal laser scanning microscope (FV1200, Olympus, Japan) equipped with an Ar/Hg laser.

### 2.12. Encapsulation Efficiency (EE) and Encapsulation Yield (EY)

The encapsulation efficiency (EE) (%) and encapsulation yield (EY) (%) were determined following the method described by Bakry et al. [28] and Martin et al. [29] with slight modifications. Firstly, encapsulated LGG particles (0.1 g) were washed with sterile saline and centrifuged at 2504× *g* for 10 min. The supernatant was serially diluted in sterile saline and coated onto MRS agar plates. After incubation at 37 °C for 48 h under anaerobic conditions, the colonies of LGG were counted, representing the number of free LGG on the surface of the encapsulated particles (S). Next, the encapsulated particles were dispersed in 10 mL of sterilized phosphate buffer (0.1 M, pH 7.0) after washing with sterile saline. They were then incubated at 37 °C and 200 rpm for 2 h to release the encapsulated LGG internally.

The number of viable LGG released from the encapsulated particles (R) was determined by the plate counting method. The sum of S and R represented the total number of viable LGG in the encapsulated particles (T). The encapsulation efficiency and encapsulation yield were calculated using the following equations:

(1) $$EE\;(\%)=\frac{R}{T}\times 100$$

(2) $$EY\;(\%)=\frac{T}{W}\times 100$$

where R is the number of viable LGG released from the encapsulated particles, S is the number of free LGG on the surface of the encapsulated particles, T is the total number of viable LGG in the encapsulated particles, and W is the initial number of LGG added before encapsulation.

### 2.13. Tolerance of Encapsulated LGG Under Simulated Gastrointestinal Conditions

To evaluate the tolerance of the encapsulated LGG particles and free LGG in simulated gastric juice (SGJ) and simulated intestinal fluid (SIJ), the protocols depicted by Bi et al. [30] and Ren et al. [31] were followed. In brief, 0.1 g of encapsulated LGG particles was dispersed in 10 mL of SGJ (0.9% NaCl, 3 g/L pepsin, pH 2.0). The mixture was then incubated at 37 °C and 120 r/min for varying periods of time: 0 h, 0.5 h, 1 h, and 2 h. The viable bacteria were quantified at each point using MRS agar. After that, the gastric digesta precipitate was collected through centrifugation at 4 °C and 7100× *g* for 5 min and added to SIJ, which contained 10 g/L bile salt and 3 g/L pancreatic enzyme dissolved in a 100 mM phosphate buffer at pH 7.4. The viable bacteria were collected after 1 h, 2 h, and 4 h of digestion at 37 °C and 120 r/min, and the colonies of LGG were estimated using the plate counting method. The survival rate (SR) was calculated using the following equation:

(3) $$SR\;(\%)=\frac{N}{T}\times 100$$

where N is the survival number of LGG from the encapsulated particles, and T is the total number of viable LGG in the encapsulated particles.

### 2.14. Storage Stability

To assess the stability of the encapsulated particles during storage, the encapsulated particles were stored at 4 °C and −20 °C for a period of 5 weeks. The viable LGG count was determined on a weekly basis.

### 2.15. Statistical Analysis

All experiments were independently conducted at least three times. Analysis of variance (ANOVA) was performed using DPS 7.05 software (Wiley-Blackwell, Hoboken, NJ, USA), and Duncan’s test was utilized with a significance level of *p* < 0.05.

## 3. Results and Discussion

### 3.1. Amylose and RS Content

Table 1 presents the amylose and RS contents of UDBS and the debranched lentil starch samples. Among them, UDBS exhibited the lowest amylose (23.20%) and RS contents (13.11%). In contrast, the different debranched starch (DBS30, DBS60, and DBS90) samples showed significantly higher proportions of amylose and RS. This suggested that the debranching treatment with pullulanase promoted the rearrangement of starch molecules and enhanced the enzymatic resistance of UDBS. An increase in amylose content can be attributed to the enzymatic hydrolysis of the α-1,6 glucosidic linkages at the terminal points of amylopectin by pullulanase. This enzymatic action leads to the formation of more linear amylose chains and subsequently results in a higher amylose content. Notably, the amylose and RS contents of the debranched starch increased with the enzyme addition level, reaching their peak in DBS90 (amylose content > 75%, resistant starch > 23%), which was markedly higher than DBS30 and UDBS. The finding aligned with the that of Shi et al. [24], who documented a similar increase in the degree of debranching and RS content with pullulanase addition. Zhao et al. [32] also demonstrated that the pullulanase-debranched starch exhibited better thermal stability and a higher content of resistant starch in comparison to natural starch. The retrograded RS originates from linear amylose molecules. These molecules tend to reassociate as double helices after cooking and cooling. The debranched starch, characterized by shorter linear chains, displays enhanced molecular mobility. This property allows the chains to readily entangle, rearrange, and aggregate via hydrogen bonding during low-temperature recrystallization, transiting from an irregularly defined to a well-defined crystalline structure. Research has consistently shown that starches with higher amylose content lead to increased levels of RS [33,34,35].

### 3.2. Molecular Weight Distribution by HPSEC

The *M_w_* and PDI (polydispersity index) values of UDBS and the debranched lentil starch samples are displayed in Table 1. The *M_w_* value of UDBS was 2.676 × 10^7^ g/mol, while the debranched starches (DBS30, DBS60, and DBS90) had significantly lower values ranging from 4.070 × 10^5^ to 7.511 × 10^5^ g/mol, with variations among the debranched starches at different degrees of debranching. The molecular weight of linear amylose (molecular weight ≈ 10^6^) is generally lower than that of the branched amylopectin (molecular weight ≈ 10^8^) [27,36,37]. According to Liu et al. [38], debranched starch (DBS) is modified by debranching enzymes (pullulanase) that selectively hydrolyze 1,6-α-d-glycosidic bonds, leading to the formation of linear short-chain molecules of low molecular weights. Moreover, increasing the pullulanase addition led to increased levels of amylose and resistant starch, while reducing the *M_w_* and PDI values. The PDI value characterizes the homogeneity of the molecular weight distribution, with a value closer to 1 indicating a more even distribution [26]. Thus, the lower *M_w_* and more uniform distribution of amylose molecules facilitated their efficient arrangement and stacking through hydrogen bonding, leading to a more ordered and enzymatically resistant molecular structure. These results align with findings reported by Liu et al. [39].

### 3.3. Crystallinity by XRD

**Figure 2:**
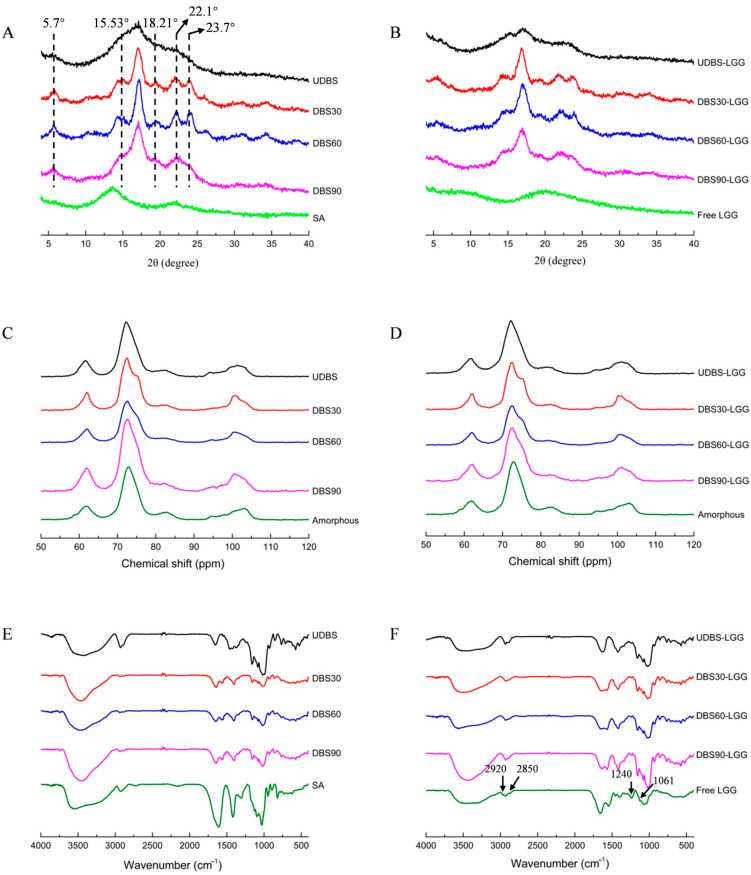
X-ray diffraction pattern of (**A**) UDBS, DBS, and (**B**) encapsulated LGG particles; ^13^C NMR spectra of (**C**) UDBS, DBS, and (**D**) encapsulated LGG particles; Fourier-transform infrared spectroscopy of (**E**) UDBS, DBS, and (**F**) encapsulated LGG particles. UDBS: undebranched starch; DBS30: debranched lentil starch prepared with the addition of pullulanase at a concentration of 30 NPUN/g; DBS60: debranched lentil starch prepared with the addition of pullulanase at a concentration of 60 NPUN/g; DBS90: debranched lentil starch prepared with the addition of pullulanase at a concentration of 90 NPUN/g; UDBS-LGG: UDBS-based encapsulated LGG particles; DBS30-LGG: DBS30-based encapsulated LGG particles; DBS60-LGG: DBS60-based encapsulated LGG particles; DBS90-LGG: DBS90-based encapsulated LGG particles; SA: sodium alginate; Free LGG: free *Lacticaseibacillus rhamnosus* GG; Amorphous: amorphous starch.

Figure 2A,B show the X-ray diffraction patterns of the DBS samples and DBS-based encapsulated particles. The UDBS sample exhibited strong diffraction peaks at 2θ = 15.53°, 17.25°, and 23.2°, as well as weak peaks at 2θ = 5.7° and 18.21°, suggesting a characteristic C-type crystalline structure. In contrast, the debranched starch samples (DBS30, DBS60, and DBS90) were identified as typical B-type crystal polymorphs, with doublet peaks observed at 2θ = 17.3°, 22.1°, 23.7°, and tiny peaks at 2θ = 5.7° and 15.5° [40,41].

The X-ray pattern indicated a loss of crystallinity for UDBS in comparison to that for native lentil starch [31]. For UDBS, the gelatinization process generally involves the reduction in crystalline regions in the starch granules by disrupting the presence of naturally occurring resistant starch. While this process increases starch chain mobility, there is a slight possibility of retrograded RS formation, as only the amyloses with optimal chain lengths are preferable for molecular rearrangement. In contrast, during the pullulanase debranching process for DBS, the heating process destroys naturally occurring RS, but an enhanced yield of retrograded RS can be achieved due to enzymatic debranching. This enzymatic process generates a higher proportion of short amylose and amylopectin chains with appropriate lengths, facilitating greater chances for amylose association into double helices and enabling further molecular realignment or aggregation into a new crystalline structure during low-temperature retrogradation. Numerous studies have demonstrated that starches with higher amylose content lead to increased levels of RS [33,34,35]. Previous reports have highlighted the significant influence of specific conditions on the formation of type A and type B crystal structures in starch. Specifically, a high substrate level, high recrystallization temperature, and the presence of short linear chains of starch were found to favor the development of the type A crystalline structure. Conversely, low substrate levels, low recrystallization temperatures, and long linear chains were observed to promote the development of the type B crystalline polymorph [42,43,44]. Simultaneously, an additional peak at 2θ = 19.8° was also observed, indicating the presence of a type V crystal polymorph in the debranched starch. This may be attributed to the amylose chains produced by autoclaving and the debranching treatment of lentil starch, which rearranged to form crystallites characteristic of single helices [27]. According to Table 2, the relative crystallinity (C1) of different samples, as calculated by XRD patterns, followed the order: DBS60 > DBS90 > DBS30 > UDBS, which was consistent with the trend in diffraction peak intensity on the X-ray diffractogram. The C1 value of DBS90 was lower than that of DBS60, possibly due to the shorter debranched starch chain caused by a greater addition of pullulanase. This resulted in the reorganization of starch chains into microcrystals, hindering the organization of perfectly ordered starch crystals during recrystallization and leading to a relatively weak long-range ordered structure as observed in XRD [24].

As depicted in Figure 2A,B, sodium alginate (SA) and LGG exhibited no crystalline structure on their XRS spectra. The XRD pattern of DBS-based encapsulated particles resembled that of the debranched starch samples, displaying a characteristic B+V crystalline pattern, although the diffraction peak intensity was noticeably reduced in the encapsulated particles. This observation aligns with the considerably lower relative crystallinity (C1) as indicated in Table 2.

### 3.4. Structural Conformation by ^13^C CP/MAS NMR

The ^13^C CP/MAS NMR spectra of different DBS samples and encapsulated LGG particles are presented in Figure 2C,D. The calculated crystallinity (C2), double-helical level, single-helix content, and PPA are summarized in Table 2. Notably, while the results for the double helix did not exhibit significant differences, the C2 values in UDBS samples were considerably lower than in DBS samples. This may be ascribed to the presence of long starch chains in unbranched starch, which hindered the development of a well-organized crystal structure following double helix formation. In contrast, debranched starch with shorter linear chains and a lower molecular weight demonstrates enhanced molecular mobility; these chains, when exposed to low-temperature recrystallization, could readily entangle, rearrange, and aggregate via hydrogen bonding, causing the transition from an irregularly constructed state to a well-defined crystalline structure [45]. Furthermore, among the DBS samples examined, DBS60 and DBS90 showed the highest level of relative crystallinity (C2), followed by DBS30. Cai et al. [46] demonstrated that debranched starch with a narrow molecular weight distribution, characterized by a low DPI nearing 1, significantly enhances the formation of a highly organized crystalline structure. Conversely, debranched starch with a wide molecular weight distribution favors the development of crystal structure with low crystallinity. This conclusion aligns with the molecular weight distribution results obtained in this study (Table 1), suggesting that the evolution of the relative crystallinity (C2) of the debranched starch samples follows a trend opposite to that of the DPI values. As shown in Table 2, the DBS samples exhibited a higher double-helix content compared to their corresponding relative crystallinity values, and the C2 values derived from ^13^CNMR were higher than the C1 values calculated from the XRD spectra. This observation was supported by Barros et al. [47], who showed that not all double helices are found exclusively within the crystalline region; some are distributed in the amorphous region. While XRD is capable of identifying only the repeating structures of double helices present in starch, solid-state ^13^CNMR can monitor the partially intact three-dimensional crystalline organization, including sub-crystalline regions, overlapping crystalline and amorphous regions, along with double helices aligned in amorphous domains [48]. The ^13^C NMR spectra of the debranched starches also revealed the presence of a single helix, which exhibited an increased trend upon the addition of pullulanase. This corresponded with the presence of V-type crystalline peaks on the XRD patterns. The PPA value displayed an inverse trend to the C2 value, with the highest PPA value observed in DBS30. The C2 values of the encapsulated LGG particles followed a similar order to that of their corresponding debranched starch samples. Compared to the debranched starch, the encapsulated LGG particles exhibited a rise in the amount of double-helix structure and a reduction in C2 value. This can be ascribed to the effective encapsulation of debranched starch with sodium alginate, which hindered the further aggregation and crystalline structure formation among the double helices.

The C2 values of the encapsulated LGG particles followed the same order as their corresponding debranched starch: DBS90-LGG > DBS60-LGG > DBS30-LGG > UDBS-LGG. Additionally, the encapsulated LGG particles exhibited a higher level of double-helical structure, while the C2 value tended to decrease compared to DBS. This indicated that the wrapping of debranched starch around the external surface of sodium alginate inhibits the further aggregation of adjacent double helices, thereby preventing the formation of a crystalline structure. The observation aligns with the previous results of Zhang et al. [49], who found a reduction in crystallinity for β-carotene microcapsules prepared with octenyl succinic anhydride-modified starch and trehalose. A similar discovery has also been reported in a previous study [50].

### 3.5. Molecular Order by FT-IR

The FT-IR spectra of the DBS samples are presented in Figure 2E. The broad absorption band between 3000 and 3600 cm^−1^ represents the stretching vibrations of the hydroxyl groups, which correspond to the differences in the intra- or inter-molecular hydrogen bonding forces of starch molecules [27]. The absorptions at 1047 cm^−1^ and 995 cm^−1^ are associated with molecular order, while the IR band at 1022 cm^−1^ indicates the presence of an amorphous or disordered region of starch granules. The ratios of 995/1022 cm^−1^ and 1047/1022 cm^−1^ can thus be utilized to quantify the degree of double helicity (DD) and the degree of order (DO), respectively [40]. Table 2 demonstrates that the DD and DO values of debranched starch increased with an increased addition of pullulanase, consistent with the change in absorption intensity between 3000 and 3600 cm^−1^. This observation aligns with the varying trend of C2 values calculated by ^13^C NMR for different debranched starches.

The absorption band at ~3400 cm^−1^ on the FT-IR spectra of free LGG as shown in Figure 2F indicates the stretching vibration of hydroxyl groups. Furthermore, the absorptions at 2920 cm^−1^ and 2850 cm^−1^ correspond to the characteristic asymmetric stretching vibrations of methyl and methylene groups, respectively, which may not be a distinctive peak of LGG. Additionally, the absorption bands observed at 1240 cm^−1^ and 1061 cm^−1^ were linked to the P=O group in the phosphate backbone and the C-O-C group in the cytoderm component, respectively [51]. The debranched starch-based encapsulated particles exhibited a comparable FT-IR pattern to that of debranched starch, with only a few discernible peaks of SA and LGG. This finding implies that the debranched starch effectively encapsulated the external sodium alginate and LGG within it. A comparable finding was documented by Zaeim et al. [52]. Previous studies have also consistently shown that the distinctive absorption bands of encapsulated substances within the encapsulated particles were noticeably diminished in their FT-IR spectra, providing further evidence of successful encapsulation.

### 3.6. Morphological Characteristics by SEM

**Figure 3:**
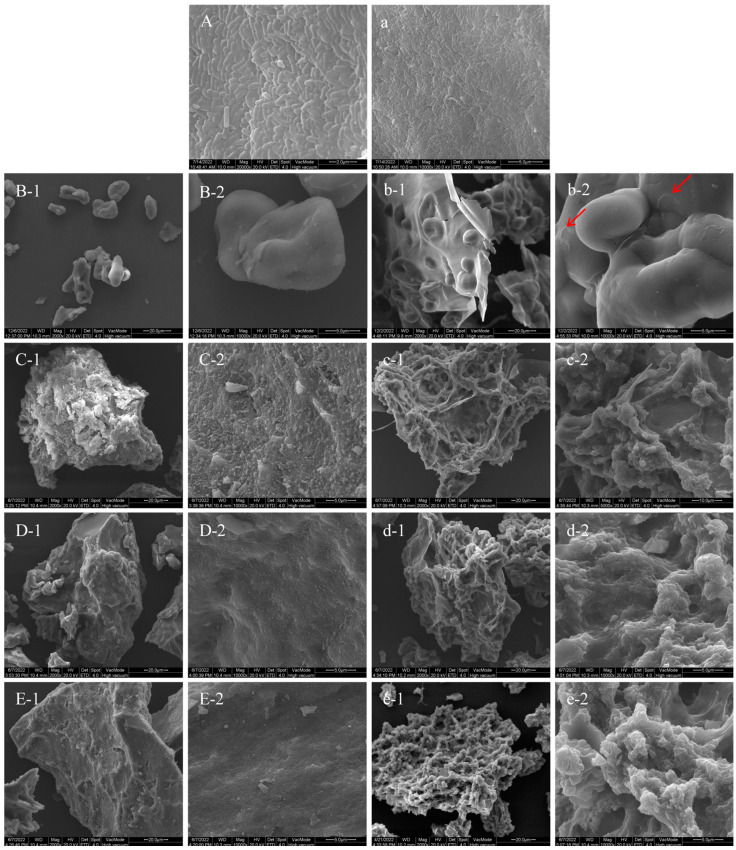
Scanning electron micrographs of UDBS, DBS, and encapsulated LGG particles. (**A**): *Lacticaseibacillus rhamnosus* GG (×20,000); (**a**): *Lacticaseibacillus rhamnosus* GG (×10,000); (**B-1**): undebranched starch (UDBS) (×2000); (**B-2**) undebranched starch (UDBS) (×10,000); (**b-1**): UDBS-based encapsulated LGG particles (×2000); (**b-2**) UDBS-based encapsulated LGG particles (×10,000); (**C-1**): debranched lentil starch prepared with the addition of pullulanase at a concentration of 30 NPUN/g (DBS30) (×2000); (**C-2**): debranched lentil starch prepared with the addition of pullulanase at a concentration of 30 NPUN/g (DBS30) (×10,000); (**c-1**): DBS39-based encapsulated LGG particles (×2000); (**c-2**) DBS39-based encapsulated LGG particles (×10,000); (**D-1**): debranched lentil starch prepared with the addition of pullulanase at a concentration of 60 NPUN/g (DBS60) (×2000); (**D-2**): debranched lentil starch prepared with the addition of pullulanase at a concentration of 60 NPUN/g (DBS60) (×10,000); (**d-1**): DBS60-based encapsulated LGG particles (×2000); (**d-2**): DBS60-based encapsulated LGG particles (×10,000); (**E-1**): debranched lentil starch prepared with the addition of pullulanase at a concentration of 90 NPUN/g (DBS90) (×2000); (**E-2**): debranched lentil starch prepared with the addition of pullulanase at a concentration of 90 NPUN/g (DBS90) (×10,000); (**e-1**): DBS90-based encapsulated LGG particles (×2000); (**e-2**) DBS90-based encapsulated LGG particles (×10,000). The red arrow indicates the presence of LGG.

Figure 3 depicts the morphological characteristics of UDBS, the debranched starch samples (DBS30, DBS60, and DBS90), and the encapsulated LGG particles as captured by SEM. The general size of lentil starch granules observed in this study was in the range of 10–20 μm, similar to that reported in previous studies [53,54]. The surface of native lentil starch granules appears much smoother [27]. However, following heat treatment, the starch granule surfaces underwent deformation, displaying distinct folds and bonding between granules, resulting in a non-spherical shape. The gelatinization and cooling processes likely contributed to adhesion between disintegrated starch granules, leading to agglomeration formation. The starch granular structure was further destroyed after being treated with pullulanase, resulting in the entanglement and agglomeration of DBS molecules during recrystallization. SEM images of DBS30, DBS60, and DBS90 displayed an irregular and compact block structure with a rough surface. These images also exhibited layered strips, grooves, and recrystallized fragments. As the amount of pullulanase increased, the structure of the debranched starch became more compact, and fewer surface fragments were observed. This provides further evidence for the formation of a more ordered internal arrangement of debranched starch, consistent with the observations from the FT-IR and ^13^C NMR analyses. Similar findings were observed previously [21].

It can be seen from Figure 3b–e that the encapsulated particles showed increased roughness as compared with the respective DBS material. In addition, the encapsulated LGG particles clearly experienced coalescence during encapsulation. The SEM image suggested that undebranched starch did not fully encapsulate sodium alginate (Figure 3b). Instead, some of the sodium alginate remained exposed, and a small amount of LGG was present on the surface layer of the encapsulated particles. The partial encapsulation observed implies a somewhat restricted encapsulation effect. In contrast, when debranched starch was employed as the encapsulating wall material, the SEM images indicated that the sodium alginate was completely covered and the LGG was well encapsulated within the encapsulated particles. Among these, DBS60-LGG and DBS90-LGG exhibited a relatively more compact structure, indicating their potential to provide enhanced protection for the encapsulates. Hassan et al. [55] also found that the prebiotic microcapsules encapsulated with a combination of alginate and resistant starch exhibited a more compact structure without any cracks, compared to microcapsules using only alginate as the wall material.

### 3.7. Morphological Characteristics by CLSM

**Figure 4:**
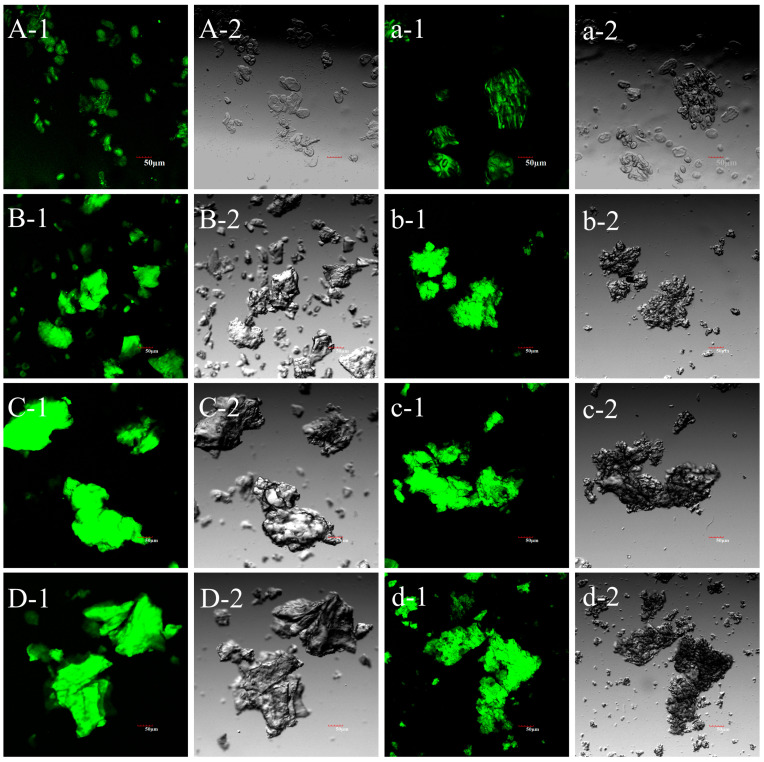
CLSM images of UDBS, DBS, and encapsulated LGG particles. (**A-1**): undebranched starch (UDBS) (bright field); (**A-2**): undebranched starch (UDBS) (dark field); (**B-1**): debranched starch with the addition of pullulanase in the amount of 30 NPUN/g (DBS30) (bright field); (**B-2**): debranched starch with the addition of pullulanase in the amount of 30 NPUN/g (DBS30) (dark field); (**C-1**): debranched starch with the addition of pullulanase in the amount of 60 NPUN/g (DBS60) (bright field); (**C-2**): debranched starch with the addition of pullulanase in the amount of 60 NPUN/g (DBS60) (dark field); (**D-1**): debranched starch with the addition of pullulanase in the amount of 90 NPUN/g (DBS90) (bright field); (**D-2**): debranched starch with the addition of pullulanase in the amount of 90 NPUN/g (DBS90) (dark field); (**a-1**): encapsulated LGG particles based on UDBS (bright field); (**a-2**): encapsulated LGG particles based on UDBS (dark field); (**b-1**): encapsulated LGG particles based on DBS30 (bright field); (**b-2**): encapsulated LGG particles based on DBS30 (dark field); (**c-1**): encapsulated LGG particles based on DBS60 (bright field); (**c-2**): encapsulated LGG particles based on DBS60 (bright field); (**d-1**): encapsulated LGG particles based on DBS90 (bright field); (**d-2**): encapsulated LGG particles based on DBS90 (dark field).

Confocal laser scanning microscopy (CLSM) has emerged as a valuable tool for providing insights into the internal organization of starch granule morphology. The APTS fluorophore is able to efficiently and specifically label the reducing end of hemiacetal rings in starch macromolecules. In general, regions displaying relatively strong fluorescence intensity on a CLSM image indicate a high abundance of amylose or low molecular weight amylopectin fractions [56]. The CLSM images in Figure 4A–D revealed that the granular structure of UDBS remains intact, while DBS formed larger aggregates after recrystallization. Furthermore, discernible differences in the fluorescence intensity of starch samples with different degrees of debranching were observed. DBS90 and DBS60 exhibited relatively strong fluorescence intensity, followed by DBS30, while UDBS demonstrated the weakest fluorescence intensity due to its lower content of straight-chain starch. Notably, the fluorescence intensity of DBS60 and DBS90 was more evenly distributed compared to DBS30. These findings corresponded well with the *M_w_*/*M_n_* values obtained from HPSEC, suggesting that the molecular weight distribution of DBS60 and DBS90 was more homogeneous in comparison to DBS30.

The CLSM images of encapsulated LGG particles (Figure 4a–d) illustrated that the fluorescence intensity of the encapsulated particles was noticeably weaker compared to that of the debranched starch, and the fluorescence intensity exhibited a more heterogeneous distribution, forming numerous network structures on the sample surface. This phenomenon is believed to have been caused by the presence of debranched starch covering both the cavity and surface of the sodium alginate network. Figure 4a-1 clearly depicts that the UDBS-LGG encapsulated particles were inadequately encapsulated and did not efficiently envelop the sodium alginate. Additionally, the double-helical structure of the DBS60-LGG and DBS90-LGG encapsulated particles appeared to be more tightly packed, which is consistent with the SEM observation.

### 3.8. Encapsulation Efficiency (EE) and Encapsulation Yield (EY) of Encapsulated LGG Particles

Encapsulation efficiency (EE) and encapsulation yield (EY) are commonly used indicators for evaluating the performance of encapsulated LGG particles. A higher EE value indicates a larger proportion of the core material being encapsulated within the wall material matrix while minimizing the presence of core material adhering to the surface of the encapsulated particles. Similarly, a higher EY value signifies a greater quantity of the original core material being encapsulated [28]. Chelating agents, such as phosphoric, lactic, and citric acids, have a strong affinity for calcium ions, rendering calcium alginate-based encapsulated particles prone to disintegration, ultimately leading to the release of *Lacticaseibacillus rhamnosus*. The EE and EY values of UDBS-LGG, DBS30-LGG, DBS60-LGG, and DBS90-LGG were calculated and are summarized in Table 3. The EE (69.59%) and EY (36.49%) values of undebranched starch were the lowest, indicating the inadequate encapsulation of LGG within the encapsulated particles, consistent with the SEM results. In contrast, debranched starch showed improved encapsulation efficacy for LGG, with relatively fewer free bacteria observed on the surface of the encapsulated particles. The EE and EY values of DBS60-LGG and DBS90-LGG were significantly higher compared to DBS30-LGG. Based on the work of Sun et al. [57], the formation of encapsulated particles occurs in two main stages. Initially, double helices associate and clusters form. This is followed by the rearrangement of these clusters into encapsulated particles. The DBS60 and DBS90 samples show increased levels of flexible linear amylose and a low molecular weight (Table 1); these flexible chains facilitate both helix alignment and intermolecular reassociations, leading to improved dispersibility and structural stability [24]. This makes them suitable for use as encapsulation wall materials, which is evident from their higher EE and EY values in comparison to DBS30. This is also reflected in their higher C1 and C2 values, suggesting that during retrogradation, a higher proportion of short amylose chains enhances the association of amylose into double helices and promotes further aggregation into a crystalline structure.

### 3.9. The Tolerance of Encapsulated LGG Under Simulated Gastrointestinal Conditions

**Figure 5:**
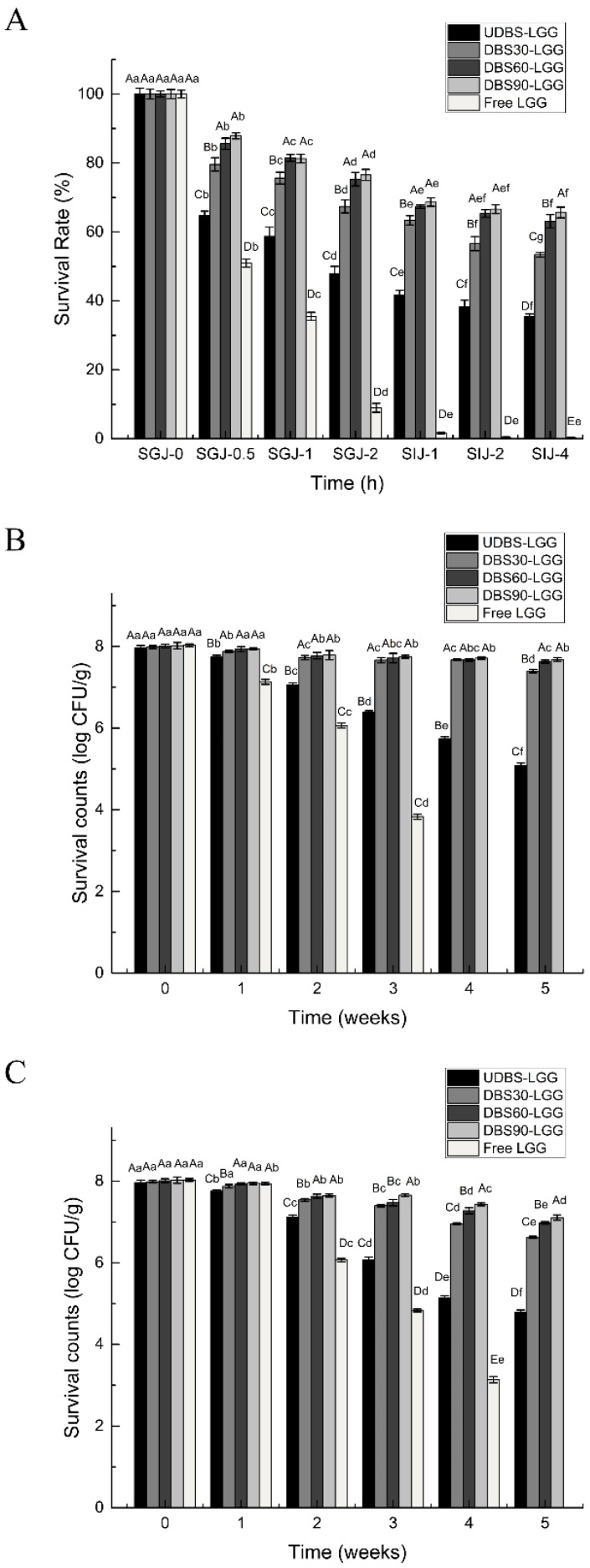
(**A**) Survival rate of free LGG and encapsulated LGG particles under simulated gastrointestinal juice in vitro; the storage stability of free LGG and encapsulated LGG particles at (**B**) 4 °C and (**C**) −20 °C. UDBS-LGG: UDBS-based encapsulated LGG particles; DBS30-LGG: DBS30-based encapsulated LGG particles; DBS60-LGG: DBS60-based encapsulated LGG particles; DBS90-LGG: DBS90-based encapsulated LGG particles; Free LGG: free *Lacticaseibacillus rhamnosus* GG. For samples stored for the same time duration (0, 1, 2, 3, 4, or 5 weeks), mean values bearing different capitalized letters are significantly different (*p* < 0.05) as per Duncan’s multiple comparison test. For the same sample, mean values bearing different lower-case letters are significantly different (*p* < 0.05) as per Duncan’s multiple comparison test.

For probiotics to effectively deliver their beneficial effects, it is crucial for them to maintain their viability while passing through the gastrointestinal tract. The efficacy of the encapsulation system in safeguarding LGG can be evaluated through the measurement of encapsulated LGG particle survival rates in SGJ and SIJ in vitro. Table 4 presents the reference count for calculating the survival rate of encapsulated LGG particles. As depicted in Figure 5A, the survival rate of free LGG significantly decreased when exposed to simulated gastric juice. Specifically, the initial survival rate in simulated gastric juice decreased by half after 0.5 h (SGJ-0.5 h), and further dropped to only 8.99% after 2 h of incubation. In contrast, the survival rates of UDBS-LGG, DBS30-LGG, DBS60-LGG, and DBS90-LGG in SGJ for 2 h (SGJ-2 h) were 47.82%, 67.42%, 75.31%, and 76.56%, respectively. In simulated intestinal juice, the free LGG was nearly completely inactivated after 4 h (SIJ-4 h), while the survival rates of UDBS-LGG, DBS30-LGG, DBS60-LGG, and DBS90-LGG at SIJ-4 h were 35.44%, 53.42%, 63.06%, and 65.63%, respectively. Notably, DBS60-LGG and DBS90-LGG exhibited significantly superior protective effects for LGG compared to UDBS-LGG and DBS30-LGG. The decline in encapsulated particle survival rates was relatively more pronounced at SGJ-0.5 h, likely due to the inactivation of free LGG present on the surfaces of the encapsulated particles when exposed to simulated gastric fluid. Subsequently, the survival rate of the encapsulated particles in simulated gastric fluid experienced a relatively minor decrease and gradually stabilized, providing substantial protection against LGG’s intolerance to the gastrointestinal environment.

The incorporation of debranched starch into sodium alginate gels during the encapsulated particle formation process facilitated the development of polymeric networks, enabling successful encapsulation of LGG and its partial isolation from environmental conditions [58]. de Araújo Kailasapathy et al. [59] also found that the addition of starch to alginate could enhance the survival of probiotic bacteria in acidic environments. Furthermore, a separate study demonstrated the highest survival rates (with a loss rate of less than 1 log CFU/g) when bacteria were encapsulated in starch–alginate beads [60].

### 3.10. Storage Stability of Encapsulated LGG Particles

Probiotic encapsulated particles should have an extended shelf life, making storage stability a critical factor in assessing their performance. The storage stability was evaluated by monitoring the viable LGG count weekly, with samples collected at specific intervals to assess probiotic activity. The viability of the encapsulated particles and free LGG was assessed during long-term storage at 4 °C and −20 °C, as depicted in Figure 5B,C. The count of free LGG dropped rapidly and became completely inactive starting from the second week. In contrast, the number of viable bacteria in the encapsulated particles gradually decreased as the storage time increased. However, it was observed that storing the encapsulated particles at a temperature of −20 °C resulted in comparatively lesser damage to the encapsulated particles. At 4 °C, UDBS-LGG showed the highest decrease (3.17 log CFU/g), followed by DBS30-LGG (1.36 log CFU/g), while DBS90-LGG demonstrated the most effective protection for LGG, with a decrease of 0.92 log CFU/g. At −20 °C, in comparison to UDBS-LGG and DBS30-LGG, both DBS60-LGG and DBS90-LGG exhibited better storage stability, with decreases of 0.38 and 0.34 log CFU/g, respectively. These findings suggested that the storage stability of the encapsulated particles was affected by factors such as molecular order, crystallinity, and the compact structure of the debranched starches. It seemed that a higher degree of molecular order, higher values of crystallinity, and a more compact structure contribute to improved storage stability. Godward et al. [61] conducted a similar study while investigating probiotic encapsulated particles, and their findings demonstrated that the encapsulated particles could maintain high viability when stored at 8 °C. Similarly, Etchepare et al. [62] used alginate and resistant starch (Hi maize) to produce encapsulated Lactobacillus acidophilus particles. These encapsulated particles retained viability for a minimum of 30 days in a freeze-dried form when stored at room temperature and for at least 135 days in a moist form.

## 4. Conclusions

The encapsulated LGG particles were prepared using debranched starch as the encapsulation wall material with varying degrees of debranching. Upon recrystallization, the crystal polymorph of starch transformed from C- to B+V-type crystals. Moreover, the molecular weight of the debranched starch decreased, while the contents of amylose and resistant starch, along with the relative crystallinity, showed a substantial increase. Increasing the addition of pullulanase led to a decrease in the molecular weight and polydispersity index of the lentil starch. This led to a more homogenous molecular distribution while simultaneously increasing the amylose content, and crystallinity also increased. This also contributed to a more orderly and compact structure. Compared to UDBS-LGG and DBS30-LGG, DBS60-LGG and DBS90-LGG exhibit higher encapsulation efficiency and encapsulation yield. This leads to enhanced protection for LGG in simulated gastrointestinal conditions and storage processes. The findings suggest that debranched starches with higher degrees of debranching exhibit a more homogeneous molecular weight distribution, a more ordered molecular structure, increased crystallinity, and a more compact structure. As a result, these starches are capable of encapsulating larger quantities of LGG and providing superior protection for the probiotic.

## Figures and Tables

**Table 1:** Molecular weight distribution, amylose, and resistant starch content of undebranched lentil starch and debranched lentil starch with varying debranching degrees.

Samples	Amylose Content (%)	Resistant Starch Content (%)	*M_w_* (g/mol)	*M_w_*/*M_n_*
UDBS	23.20 ± 0.66 ^d^	13.11 ± 0.02 ^c^	2.676 × 10^7^ (±0.654%) ^d^	2.077 (±0.813%) ^d^
DBS30	50.25 ± 0.81 ^c^	20.63 ± 0.13 ^b^	7.511 × 10^5^ (±0.215%) ^c^	1.963 (±0.843%) ^c^
DBS60	65.32 ± 0.49 ^b^	23.79 ± 0.83 ^a^	6.633 × 10^5^ (±0.248%) ^b^	1.809 (±0.819%) ^b^
DBS90	75.44 ± 0.37 ^a^	23.30 ± 0.34 ^a^	4.070 × 10^5^ (±0.394%) ^a^	1.619 (±1.013%) ^a^

*M_w_*: weight average molecular weight; *M_w_*/*M_n_*: polydispersity index; UDBS: undebranched starch; DBS30: debranched lentil starch prepared with the addition of pullulanase at a concentration of 30 NPUN/g; DBS60: debranched lentil starch prepared with the addition of pullulanase at a concentration of 60 NPUN/g; DBS90: debranched lentil starch prepared with the addition of pullulanase at a concentration of 90 NPUN/g. Values in the same column with different lower-case letters are significantly different from each other (*p* < 0.05).

**Table 2:** Multi-scale structural parameters of UDBS, DBS, and encapsulated LGG particles.

Samples	DO Value by FT-IR	DD Value by FT-IR	Crystallinity by XRD (C1, %)	Crystallinity by ^13^C NMR (C2, %)	Double-Helix Content by ^13^C NMR (%)	Single-Helix Content by ^13^C NMR (%)	PPA by ^13^C NMR (%)
UDBS	1.0313 ± 0.0003 ^b^	1.0030 ± 0.0001 ^f^	14.26 ± 0.37 ^f^	22.36 ± 0.04 ^e^	66.06 ± 0.01 ^ab^	1.42 ± 0.04 ^a^	4.93 ± 0.06 ^bc^
DBS30	1.0164 ± 0.0001 ^f^	1.0045 ± 0.0001 ^e^	27.71 ± 0.69 ^c^	36.14 ± 0.67 ^b^	64.12 ± 0.07 ^bc^	0.71 ± 0.00 ^d^	5.09 ± 0.07 ^ab^
DBS60	1.0221 ± 0.0001 ^d^	1.0121 ± 0.0002 ^c^	37.42 ± 0.57 ^a^	43.40 ± 1.09 ^a^	64.48 ± 0.30 ^bc^	0.71 ± 0.06 ^d^	2.39 ± 0.04 ^g^
DBS90	1.0258 ± 0.0008 ^c^	1.0177 ± 0.0005 ^b^	30.84 ± 0.18 ^b^	44.23 ± 0.24 ^a^	64.61 ± 0.25 ^bc^	0.92 ± 0.02 ^c^	3.58 ± 0.20 ^f^
UDBS-LGG	1.0170 ± 0.0003 ^f^	1.0129 ± 0.0005 ^c^	11.54 ± 0.76 ^g^	18.51 ± 0.32 ^f^	63.39 ± 2.91 ^c^	1.05 ± 0.02 ^b^	4.57 ± 0.02 ^d^
DBS30-LGG	1.0200 ± 0.0003 ^e^	1.0060 ± 0.0002 ^d^	23.61 ± 0.37 ^d^	27.15 ± 1.22 ^d^	65.40 ± 0.11 ^abc^	0.71 ± 0.03 ^d^	4.22 ± 0.04 ^e^
DBS60-LGG	1.0194 ± 0.0010 ^e^	1.0045 ± 0.0015 ^e^	24.36 ± 0.44 ^d^	29.33 ± 0.52 ^c^	65.34 ± 0.01 ^abc^	0.66 ± 0.01 ^d^	5.28 ± 0.03 ^a^
DBS90-LGG	1.0390 ± 0.0390 ^a^	1.0267 ± 0.0005 ^a^	22.01 ± 0.10 ^e^	37.63 ± 1.92 ^b^	67.24 ± 1.14 ^a^	0.49 ± 0.18 ^e^	4.82 ± 0.28 ^c^

DO: degree of order (1047/1035 cm^−1^); DD: degree of double helix (986/1035 cm^−1^); PPA: proportion of amorphous phase; UDBS: undebranched starch; DBS30: debranched lentil starch prepared with the addition of pullulanase at a concentration of 30 NPUN/g; DBS60: debranched lentil starch prepared with the addition of pullulanase at a concentration of 60 NPUN/g; DBS90: debranched lentil starch prepared with the addition of pullulanase at a concentration of 90 NPUN/g; UDBS-LGG: UDBS-based encapsulated LGG particles; DBS30-LGG: DBS30-based encapsulated LGG particles; DBS60-LGG: DBS60-based encapsulated LGG particles; DBS90-LGG: DBS90-based encapsulated LGG particles. Values in the same column with different lower-case letters are significantly different from each other (*p* < 0.05).

**Table 3:** The specific count of encapsulated probiotics, the encapsulation efficiency (EE), and the encapsulation yield (EY) of encapsulated LGG particles.

Samples	Specific Count of Enpasulated Probiotics (log CFU/g)	EE (%)	EY (%)
UDBS-LGG	8.72 ± 0.02 ^c^	69.59 ± 0.60 ^c^	36.49 ± 2.16 ^c^
DBS30-LGG	8.99 ± 0.01 ^b^	92.26 ± 0.30 ^b^	68.88 ± 0.72 ^b^
DBS60-LGG	9.04 ± 0.01 ^a^	94.07 ± 0.38 ^a^	76.64 ± 0.43 ^a^
DBS90-LGG	9.05 ± 0.01 ^a^	94.36 ± 0.15 ^a^	77.88 ± 0.43 ^a^

UDBS-LGG: UDBS-based encapsulated LGG particles; DBS30-LGG: DBS30-based encapsulated LGG particles; DBS60-LGG: DBS60-based encapsulated LGG particles; DBS90-LGG: DBS90-based encapsulated LGG particles. Values in the same column with different lower-case letters are significantly different from each other (*p* < 0.05).

**Table 4:** The reference count for calculating the survival rate of encapsulated LGG particles.

Samples	SGJ-0 (log CFU/g)	SGJ-0.5 (log CFU/g)	SGJ-1 (log CFU/g)	SGJ-2 (log CFU/g)	SIJ-1 (log CFU/g)	SIJ-2 (log CFU/g)	SIJ-4 (log CFU/g)
UDBS-LGG	7.72 ± 0.02 ^a^	7.52 ± 0.03 ^b^	7.48 ± 0.03 ^b^	7.40 ± 0.03 ^c^	7.33 ± 0.02 ^d^	7.30 ± 0.04 ^de^	7.26 ± 0.01 ^e^
DBS30-LGG	7.98 ± 0.01 ^a^	7.88 ± 0.01 ^b^	7.86 ± 0.00 ^c^	7.81 ± 0.00 ^d^	7.79 ± 0.01 ^e^	7.74 ± 0.02 ^f^	7.70 ± 0.02 ^g^
DBS60-LGG	8.04 ± 0.05 ^a^	7.98 ± 0.04 ^ab^	7.95 ± 0.05 ^b^	7.92 ± 0.02 ^bc^	7.87 ± 0.04 ^c^	7.86 ± 0.04 ^c^	7.84 ± 0.05 ^c^
DBS90-LGG	8.04 ± 0.03 ^a^	7.98 ± 0.03 ^b^	7.95 ± 0.03 ^bc^	7.92 ± 0.04 ^cd^	7.88 ± 0.03 ^de^	7.86 ± 0.03 ^e^	7.86 ± 0.03 ^e^
Free-LGG	8.12 ± 0.01 ^a^	7.83 ± 0.01 ^a^	7.67 ± 0.01 ^a^	6.07 ± 0.07 ^b^	5.34 ± 0.10 ^b^	4.90 ± 1.18 ^b^	4.85 ± 1.25 ^b^

Values in the same column with different lower-case letters are significantly different from each other (*p* < 0.05).

## Data Availability

The original contributions presented in the study are included in the article, further inquiries can be directed to the corresponding author.

## References

[1] Wang X.Y., Hu R.Q., Lin F., Yang T., Lu Y.W., Sun Z.J., Li T.Y., Chen J. (2024). *Lactobacillus reuteri* or *Lactobacillus rhamnosus* GG intervention facilitates gut barrier function, decreases corticosterone and ameliorates social behavior in LPS-exposed offspring. Food Res. Int..

[2] Wang G., Jiao T., Xu Y., Li D.Z., Si Q., Hao J.F., Zhao J.X., Zhang H., Chen W. (2020). *Bifidobacterium adolescentis* and *Lactobacillus rhamnosus* alleviate non-alcoholic fatty liver disease induced by a high-fat, high-cholesterol diet through modulation of different gut microbiota-dependent pathways. Food Funct..

[3] Jang H.R., Park H.J., Kang D., Chung H., Nam M.H., Lee Y., Park J.H., Lee H.Y. (2019). A protective mechanism of probiotic *Lactobacillus* against hepatic steatosis via reducing host intestinal fatty acid absorption. Exp. Mol. Med..

[4] Gao J., Li Y., Wan Y., Hu T., Liu L., Yang S., Gong Z., Zeng Q., Wei Y., Yang W. (2019). A novel postbiotic from *Lactobacillus rhamnosus* GG with a beneficial effect on intestinal barrier function. Front. Microbiol..

[5] Barajas-Álvarez P., González-Ávila M., Espinosa-Andrews H. (2021). Recent advances in probiotic encapsulation to improve viability under storage and gastrointestinal conditions and their impact on functional food formulation. Food Rev. Int..

[6] Akanny E., Bourgeois S., Bonhommé A., Commun C., Doleans-Jordheim A., Bessueille F., Bordes C. (2020). Development of enteric polymer-based microspheres by spray-drying for colonic delivery of Lactobacillus rhamnosus GG. Int. J. Pharm..

[7] Jacob P.L., Brugnoli B., Giudice A.D., Phan H., Chauhan V.M., Beckett L., Gillis R.B., Moloney C., Cavanagh R.J., Krumins E. (2023). Poly (*Diglycerol adipate*) variants as enhanced nanocarrier replacements in drug delivery applications. J. Colloid Interface Sci..

[8] Ab’Lah N., Yusuf C.Y.L., Rojsitthisak P., Wong T.W. (2023). Reinvention of starch for oral drug delivery system design. Int. J. Biol. Macromol..

[9] Chang R., Li M., Wang Y.F., Chen H.H., Xiao J.X., Xiong L., Qiu L.Z., Bian X.L., Sun C.R., Sun Q.J. (2019). Retrogradation behavior of debranched starch with different degrees of polymerization. Food Chem..

[10] Wang D., Zhao M., Wang Y., Mu H., Sun C., Chen H., Sun Q. (2022). Research Progress on Debranched Starch: Preparation, Characterization, and Application. Food Rev. Int..

[11] Gasiński A., Kawa-Rygielska J. (2022). Mashing quality and nutritional content of lentil and bean malts. LWT-Food Sci. Technol..

[12] Joshi M., Aldred P., McKnight S., Panozzo J.F., Kasapis S., Adhikari R., Adhikari B. (2013). Physicochemical and functional characteristics of lentil starch. Carbohydr. Polym..

[13] Thakur M., Sharma N., Rai A.K., Singh S.P. (2021). A novel cold-active type I pullulanase from a hot-spring metagenome for effective debranching and production of resistant starch. Bioresour. Technol..

[14] Martínez-Cano B., Mendoza-Meneses C.J., García-Trejo J.F., Macías-Bobadilla G., Aguirre-Becerra H., Soto-Zarazúa G.M., Feregrino-Pérez A.A. (2022). Review and Perspectives of the Use of Alginate as a Polymer Matrix for Microorganisms Applied in Agro-Industry. Molecules.

[15] Abka-khajouei R., Tounsi L., Shahabi N., Patel A.K., Abdelkafi S., Michaud P. (2022). Structures, Properties and Applications of Alginates. Mar. Drugs.

[16] Xu C., Ban Q.F., Wang W., Hou J.C., Jiang Z.M. (2022). Novel nano-encapsulated probiotic agents: Encapsulate materials, delivery, and encapsulation systems. J. Control. Release..

[17] Kwoak C.S., Kim S.J., Kim C.S. (2022). Microencapsulation of *Lactobacillus plantarum* ATCC 8014 and *Bifidobacterium bifidum* ATCC 1903 in alginate blended with starch by extrusion technique. Indian J. Anim. Sci..

[18] Thangrongthong S., Puttarat N., Ladda B., Itthisoponkul T., Pinket W., Kasemwong K., Taweechotipatr M. (2020). Microencapsulation of probiotic *Lactobacillus brevis* ST-69 producing GABA using alginate supplemented with nanocrystalline starch. Food Sci. Biotechnol..

[19] Oberoi K., Tolun A., Altintas Z., Sharma S. (2021). Effect of alginate-microencapsulated hydrogels on the survival of *Lactobacillus rhamnosus* under simulated gastrointestinal conditions. Foods.

[20] Bodjrenou D.M., Li X., Chen W., Zhang Y., Zheng B., Zeng H. (2022). Effect of Pullulanase Debranching Time Combined with Autoclaving on the Structural, Physicochemical Properties, and In Vitro Digestibility of Purple Sweet Potato Starch. Foods.

[21] Duyen T.T.M., Hung P.V. (2021). Morphology, crystalline structure and digestibility of debranched starch nanoparticles varying in average degree of polymerization and fabrication methods. Carbohydr. Polym..

[22] Hong Y., Yang J., Liu W., Gu Z., Li Z.F., Cheng L., Li C.M., Duan X.J. (2019). Sustained release of tea polyphenols from a debranched corn starch-xanthan gum complex carrier. LWT-Food Sci. Technol..

[23] Xu J., Ma Z., Ren N., Li X., Liu L., Hu X. (2019). Understanding the multi-scale structural changes in starch and its physicochemical properties during the processing of chickpea, navy bean, and yellow field pea seeds. Food Chem..

[24] Shi J., Sweedman M.C., Shi Y.C. (2018). Structural changes and digestibility of waxy maize starch debranched by different levels of pullulanase. Carbohydr. Polym..

[25] Morsy M.K., Morsy O.M., Abdelmonem M.J.A., Elsabagh R. (2022). Aanthocyanin-ccolored microencapsulation effects on survival rate of *Lactobacillus rhamnosus* GG, color Stability, and sensory parameters in strawberry nectar model. Food Bioproc. Tech..

[26] Zhou D., Ma Z., Xu J., Li X., Hu X. (2019). Resistant starch isolated from enzymatic, physical, and acid treated pea starch: Preparation, structural characteristics, and in vitro bile acid capacity. LWT-Food Sci. Technol..

[27] Yin X., Ma Z., Hu X., Li X., Boye J.I. (2018). Molecular rearrangement of Laird lentil (*Lens culinaris* Medikus) starch during different processing treatments of the seeds. Food Hydrocoll..

[28] Bakry A.M., Huang J., Zhai Y., Huang Q. (2019). Myofibrillar protein with κ- or λ-carrageenans as novel shell materials for microencapsulation of tuna oil through complex coacervation. Food Hydrocoll..

[29] Martin M.J., Lara-Villoslada F., Ruiz M.A., Morales M.E. (2013). Effect of unmodified starch on viability of alginate-encapsulated *Lactobacillus fermentum* CECT5716. LWT-Food Sci. Technol..

[30] Bi H., Xu Y., Fan F., Sun X. (2022). Effect of drying methods on *Lactobacillus rhamnosus* GG microcapsules prepared using the complex coacervation method. J. Food Sci..

[31] Ren N., Ma Z., Li X., Hu X. (2021). Preparation of rutin-loaded microparticles by debranched lentil starch-based wall materials: Structure, morphology and in vitro release behavior. Int. J. Biol. Macromol..

[32] Zhao X.H., Lin Y. (2009). The impact of coupled acid or pullulanase debranching on the formation of resistant starch from maize starch with autoclaving–cooling cycles. Eur. Food Res. Technol..

[33] Li L., Jiang H.X., Campbell M., Blanco M., Jane J.L. (2008). Characterization of maize amylose-extender (ae) mutant starches. Part I: Relationship between resistant starch contents and molecular structures. Carbohydr. Polym..

[34] Lin J.H., Wang S.W., Chang Y.H. (2009). Impacts of acid-methanol treatment and annealing on the enzymatic resistance of corn starches. Food Hydrocoll..

[35] Vasanthan T., Bhatty R.S. (1998). Enhancement of resistant starch (RS3) in amylomaize, barley, field pea and lentil starches. Starch Stärke.

[36] Buléon A., Colonna P., Planchot V., Ball S. (1998). Starch granules: Structure and biosynthesis. Int. J. Biol. Macromol..

[37] Pérez S., Bertoft E. (2010). The molecular structures of starch components and their contribution to the architecture of starch granules: A comprehensive review. Starch Stärke.

[38] Liu G., Gu Z., Hong Y., Cheng L., Li C. (2017). Structure, functionality and applications of debranched starch: A review. Trends Food Sci. Tech..

[39] Liu G.D., Hong Y., Gu Z.B., Li Z.F., Cheng L., Li C.M. (2015). Preparation and characterization of pullulanase debranched starches and their properties for drug controlled-release. RSC Adv..

[40] Ma Z., Boye J.I. (2018). Research advances on structural characterization of resistant starch and its structure-physiological function relationship: A review. Crit. Rev. Food Sci. Nutr..

[41] Lopez-Rubio A., Flanagan B.M., Gilbert E.P., Gidley M.J. (2008). A novel approach for calculating starch crystallinity and its correlation with double helix content: A combined XRD and NMR study. Biopolymers.

[42] Cai L.M., Bai Y.J., Shi Y.C. (2012). Study on melting and crystallization of short-linear chains from debranched waxy starches by in situ synchrotron wide-angle X-ray diffraction. J. Cereal Sci..

[43] Kiatponglarp W., Tongta S., Rolland-Sabaté A., Buléon A. (2015). Crystallization and chain reorganization of debranched rice starches in relation to resistant starch formation. Carbohydr. Polym..

[44] Buléon A., Véronèse G., Putaux J.L. (2007). Self-Association and crystallization of amylose. Aust. J. Chem..

[45] Haralampu S.G. (2000). Resistant starch—A review of the physical properties and biological impact of RS3. Carbohydr. Polym..

[46] Cai L.M., Shi Y.C., Rong L.X., Hsiao B.S. (2010). Debranching and crystallization of waxy maize starch in relation to enzyme digestibility. Carbohydr. Polym..

[47] Barros F., Awika J.M., Rooney L.W. (2012). Interaction of tannins and other sorghum phenolic compounds with starch and effects on in vitro starch digestibility. J. Agric. Food Chem..

[48] Gidley M.J., Bociek S.M. (1985). Molecular organization in starches: A carbon 13 CP/MAS NMR study. J. Am. Chem. Soc..

[49] Zhang L., Wei Y., Liao W.Y., Tong Z., Wang Y., Liu J.F., Gao Y.X. (2021). Impact of trehalose on physicochemical stability of β-carotene high loaded microcapsules fabricated by wet-milling coupled with spray drying. Food Hydrocoll..

[50] Liu Y.W., Xu B., An F., Liu J. (2021). Physicochemical Properties of Cassava Starch-Konjac Glucomannan Composites. Starch Stärke.

[51] Hlaing M.M., Wood B.R., McNaughton D., Ying D., Dumsday G., Augustin M.A. (2017). Effect of Drying Methods on Protein and DNA Conformation Changes in *Lactobacillus rhamnosus* GG Cells by Fourier Transform Infrared Spectroscopy. J. Agric. Food Chem..

[52] Zaeim D., Sarabi-Jamab M., Ghorani B., Kadkhodaee R., Liu W.L., Tromp R.H. (2020). Microencapsulation of probiotics in multi-polysaccharide microcapsules by electro-hydrodynamic atomization and incorporation into ice-cream formulation. Food Struct..

[53] Ma Z., Boye J.I., Simpson B.K., Prasher S.O., Monpetit D., Malcolmson L. (2011). Thermal processing effects on the functional properties and microstructure of lentil, chickpea, and pea flours. Food Res. Int..

[54] Sivakumar C., Stobbs J.A., Tu K., Karunakaran C., Paliwal J. (2024). Unravelling particle morphology and flour porosity of roller-milled green lentil flour using scanning electron microscopy and synchrotron X-ray micro-computed tomography. Powder Technol..

[55] Hassan H., Gomaa A., Subirade M., Kheadr E., St-Gelais D., Fliss I. (2020). Novel design for alginate/resistant starch microcapsules controlling nisin release. Int. J. Biol. Macromol..

[56] Roy F., Boye J.I., Simpson B.K. (2010). Bioactive proteins and peptides in pulse crops: Pea, chickpea and lentil. Food Res. Int..

[57] Sun Q., Li G., Dai L., Ji N., Xiong L. (2014). Green preparation and characterisation of waxy maize starch nanoparticles through enzymolysis and recrystallisation. Food Chem..

[58] Martín R., Olivares M., Marín M.L., Fernández L., Xaus J., Rodríguez J.M. (2005). Probiotic Potential of 3 Lactobacilli Strains Isolated From Breast Milk. J. Hum. Lact..

[59] Kailasapathy K. (2006). Survival of free and encapsulated probiotic bacteria and their effect on the sensory properties of yoghurt. LWT-Food Sci. Technol..

[60] Ta L.P., Bujna E., Antal O., Ladányi M., Juhász R., Szécsi A., Kun S., Sudheer S., Gupta V.K., Nguyen Q.D. (2021). Effects of various polysaccharides (alginate, carrageenan, gums, chitosan) and their combination with prebiotic saccharides (resistant starch, lactosucrose, lactulose) on the encapsulation of probiotic bacteria *Lactobacillus casei* 01 strain. Int. J. Biol. Macromol..

[61] Godward G., Kailasapathy K. (2003). Viability and survival of free, encapsulated and co-encapsulated probiotic bacteria in yoghurt. Milchwissenschaft.

[62] Etchepare M.D.A., Raddatz G.C., Flores E.M.D.M., Zepka L.Q., Jacob-Lopes E., Barin J.S., Grosso C.R.F., Menezes C.R.D. (2015). Effect of resistant starch and chitosan on survival of *Lactobacillus acidophilus* microencapsulated with sodium alginate. LWT-Food Sci. Technol..

